# Involvement of adenosine and standardization of aqueous extract of garlic (*Allium sativum* Linn.) on cardioprotective and cardiodepressant properties in ischemic preconditioning and myocardial ischemia-reperfusion induced cardiac injury

**DOI:** 10.1016/S1674-8301(12)60004-9

**Published:** 2012-01

**Authors:** Ashish Kumar Sharma, Arshee Munajjam, Bhawna Vaishnav, Richa Sharma, Ashok Sharma, Kunal Kishore, Akash Sharma, Divya Sharma, Rita Kumari, Ashish Tiwari, Santosh Kumar Singh, Samir Gaur, Vijay Singh Jatav, Barthu Parthi Srinivasan, Shyam Sunder Agarwal

**Affiliations:** a Department of Pharmacology, Gyan Vihar School of Pharmacy, Suresh Gyan Vihar University, Mahal, Jagatpura, Jaipur (Rajasthan) 302025, India;; b Department of Pharmacology, Delhi Institute of Pharmaceutical Sciences & Research, Pushpvihar, Sector-III. New Delhi 110017, India.

**Keywords:** *Allium sativum* Linn., ischemic preconditioning, cardioprotection, adenosine, nitrite

## Abstract

The present study investigated the effect of garlic (*Allium sativum* Linn.) aqueous extracts on ischemic preconditioning and ischemia-reperfusion induced cardiac injury, as well as adenosine involvement in ischemic preconditioning and garlic extract induced cardioprotection. A model of ischemia-reperfusion injury was established using Langendorff apparatus. Aqueous extract of garlic dose was standardized (0.5%, 0.4%, 0.3%, 0.2%, 0.1%, 0.07%, 0.05%, 0.03%, 0.01%), and the 0.05% dose was found to be the most effective. Higher doses (more than 0.05%) were highly toxic, causing arrhythmia and cardiodepression, whereas the lower doses were ineffective. Garlic exaggerated the cardioprotective effect of ischemic preconditioning. The cardioprotective effect of ischemic preconditioning and garlic cardioprotection was significantly attenuated by theophylline (1,000 µmol/L) and 8-SPT (10 mg/kg, i.p.) and expressed by increased myocardial infarct size, increased LDH level, and reduced nitrite and adenosine levels. These findings suggest that adenosine is involved in the pharmacological and molecular mechanism of garlic induced cardioprotection and mediated by the modulation of nitric oxide.

## INTRODUCTION

Myocardial infarction (MI) is a key component in the majority of cardiovascular diseases. Acute myocardial infarction (AMI), more commonly known as heart attack, is the most prevalent form of cardiovascular death in developed countries. Cardiovascular diseases account for 17 million deaths worldwide every year[1].

MI or AMI is an interruption of blood supply to a part of the heart that causes some heart cells to die. This is most commonly due to occlusion (blockage) of a coronary artery following the rupture of a vulnerable atherosclerotic plaque, which is an unstable collection of lipids and white blood cells (especially macrophages) in the wall of an artery. The resulting ischemia and oxygen shortage, if left untreated for a sufficient period of time, can cause damage or infarction of the myocardium[2].

Thus, attention has been focused on understanding the adaptive mechanism that will make the myocardium more resistant to ischemia of longer duration and to restore its viability on reperfusion. Repeated brief episodes of ischemic reperfusion have been demonstrated to make the myocardium transiently more resistant to the deleterious effect of subsequent and prolonged ischemic insult. This paradoxical form of myocardial adaptation has been termed as “ischemic preconditioning”, and is reported to limit the infarct size[3].

Ischemic preconditioning is a universal adaptive response to cellular stress. The liver, brain, and skeletal muscles, are amenable to the protective effect of preconditioning. Since stimuli other than ischemia can also produce cardioprotection, the term ischemic preconditioning has been replaced by “PRECONDITIONING” to expand its scope and applicability[4],[5].

Adenosine was the first signal transduction element identified as part of preconditioning mechanism. Adenosine is a purine nucleoside with a short *in vivo* halflife of 1.5 sec due to its rapid metabolism[6]. Unlike ATP, adenosine exists freely in cytosol of all cells and is transported in and out of the cell by a membrane transporter. Adenosine (Ado) accumulates in tissues under metabolic stress. It is not a conventional transmitter but a sort of a local hormone or in other words, a “homeostatic modulator”[7]-[10].

Adenosine receptors are members of the superfamily of G-protein-coupled receptors, with four currently recognized subtypes: A1, A2A, A2B, and A3[11]. The receptors coupled to different G-proteins and are expressed in various kinds of organs and mediate many different functions. In 1926, Dury and Szent-Gyorgyi first described the physiological effects of adenosine on cardiovascular, gastrointestinal and in renal systems[12],[13].

Adenosine is important, both as a potential trigger and also as a mediator, during the sustained ischemia. Adenosine may attenuate ischemia-reperfusion injury by a number of possible mechanisms, including purine salvaging, improved tissue perfusion, anti-inflammatory action and a direct intracellular initiator/effectors mechanism[10]. The critical role of adenosine on ischemic preconditioning is already widely accepted[14],[15].

Garlic (*Allium sativum* Linn.) belongs to the plant family Liliaceous and is a hardy perennial bulbous scapigerous herb with a flat stem[16]. Garlic is used worldwide as food additive spice and medicine. Garlic is clearly one of the most popular herbal remedies worldwide[17]. Garlic has long been used both for flavoring and for the potential benefits of preventing and curing ailments in many cultures[18]. Sanskrit records show that it was medicinally used about 5,000 y ago, and it has been used for at least 3,000 y in Chinese medicine. The Egyptians, Babylonians, Greeks, and Romans used garlic for healing purposes. Considerable anecdotal evidence supports the invaluable role of garlic as a traditional medicine in the therapy of many diseases[19]. Over the centuries, garlic has acquired a special position in the folklore of many cultures as a formidable prophylactic and therapeutic medicinal agent. It is cited in the *Egyptian Codex Ebers*, a 35-century-old document, as being useful in the treatment of heart disorders, tumors, worms, bites and other ailments. Hippocrates and Pliny the Elder were both promoters of garlic's medicinal virtues. Charak (3,000 BC), the father of Ayurvedic medicine, claimed that garlic maintains the fluidity of blood and strengthens the heart. The first-century Indian physician, Charaka, claimed that garlic acted as a heart tonic and prevented heart disease[20]. In 1858, Pasteur noted garlic's antibacterial activity, and it was used as an antiseptic to prevent gangrene during World Wars I and II[21]. Many different garlic preparations are available commercially, including freeze-dried, air-dried, essentialoil, and aged extracts. Studies have shown that garlic is beneficial in the treatment of cardiovascular diseases[1]. Over the last two decades, this important and exciting aspect of garlic has been and continues to be confirmed by basic and clinical researches reported around the world.

The chemical constituents from garlic cloves (bulbs) vary with the isolation procedure[16]. A majority of the compounds in garlic are water-soluble (97.00%) with small amounts of oil-soluble compounds also present (0.15%-0.70%) [22].

Studies have shown that garlic is beneficial in the treatment of cardiovascular diseases. It has cardioprotective properties, such as lipid-lowering, blood pressure-lowering, antioxidant (free radical scavenging, inhibition of lipid peroxidation), antiplatelet, fibrinolytic, antihypertensive, antiglycemic, antithrombotic, and antiatherogenic, procirculatory effects[17],[18],[21],[23]-[25].

Aqueous extracts of garlic inhibits platelet aggregation by reducing the formation of thromboxane, which inhibits the phospholipase activity and lipoxygenase product formation in platelets[26]. Garlic oil increases antioxidants and decreases oxidants modulated by oral application in the renal I/R (ischemia/reperfusion) injury[27]. It has been reported that garlic shows cardioprotective effect similar to ischemic preconditioning and is effective in reducing myocardial infarct size in the isolated rat heart[18].

Ischemic preconditioning and garlic have been shown to have a cardioprotective effect. So the present study is designed to investigate the role of adenosine in ischemic preconditioning and garlic cardioprotection in order to evaluate the mechanism of action and role of the adenosine receptor in garlic cardioprotection.

## MATERIALS AND METHODS

### Animals and reagents

Wistar albino rats (125-150 g) of both sexes were employed in the present study. The animals were fed on standard laboratory rat chow and had access to tap water *ad libitum*. The experimental protocol was approved by the Institutonal Animal Ethics Committee (IACE-II Gyan Vihar School of Pharmacy Reg. No. 1234/a.08/CPCSEA, under the supervision of CPCSEA, Chennai, India.

Triphenyl tetrazolium chloride was purchased from Loba-chemie (USA); heparin was gotten from Claris Life Sciences Limited, Ahemdabad, India; sodium chloride, potassium chloride, calcium chloride, magnesium sulphate, sodium bicarbonate, potassium dihydrogen phosphate, glucose, sodium nitrite, sulphanilamide, naphthylethylenediamine, and *O*-phosphoric acid were all purchased from Central Drug House, New Dehli, India; lactate dehydrogenase (LDH) Kit was bought from Merck Specialties Private Limited, Ambernath, Banglore, India; 8-(p-sulfophenyl) theophylline was purchased from Sigma-Aldrich, USA; ketamine hydrochloride injection was bought from Themis Medicare, Goregoan, Mumbai, India.

### Apparatus

Langendorff's apparatus[29] (INCO, Ambala, India), oxygen cylinder, ECG (BPL CARDIAART 108T-DIGI, New Delhi, India).

### Preparation of garlic extract

The garlic was purchased from a local market. The garlic was identified and authenticated by the botanist in the Department of Botany, University of Rajasthan, Jaipur (Rajasthan). India; voucher number RUBL 20674. Two-hundred-and-fifty grams of garlic cloves were peeled, washed, and crushed in a grinder with water in double quantity and left for overnight. The solution was first filtered with muslin cloth and then with Whatman's filter paper. This extract was added to Krebs - Henseleit's solution to produce a concentration of 0.05%[28].

### Global ischemia and reperfusion in isolated rat heart[30]

Heparin (500 U, i.p.) was administered 20 min before sacrificing the animal. The heart was then rapidly excised and immediately mounted on a Langendorff's apparatus. The aorta was retrogradely perfused at a constant pressure of 70 mm Hg with Kreb's Henseleit buffer (NaCl 118 mmol/L; KCl 4.7 mmol/L; CaCl_2_ 2.5 mmol/L; MgSO_4_· 7H_2_O 1.2 mmol/L; NaHCO_3_ 25 mmol/L; KH_2_PO_4_ 1.2 mmol/L; C_6_H_12_O_6_ 11 mmol/L), pH 7.4, maintained at 37°C bubbled with 95%O_2_ and 5%CO_2_. Flow rate was maintained between 6-9 mL/min using Hoffman's screw. The heart was enclosed by a double-walled jacket with the temperature of which was maintained by circulating water heated to 37°C. Global ischemia was produced by blocking the inflow of Krebs buffer for 20 min. It is followed by reperfusion for 40 min. ECG was monitored using two silver electrodes fixed at the left ventricular apex and right auricle. ECG was recorded immediately after stabilization, and immediately, 10 min, 20 min, 40 min after reperfusion. Coronary effluent was collected at identical intervals during reperfusion for adenosine, LDH and nitrite estimation. Coronary flow rate was also measured at the same time intervals.

### Infarct size measurement[30]

**Fig. 1:**
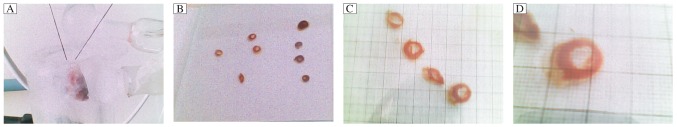
Infarct size measurement. A: Heart was mounted on Langendorff apparatus and transparent to 20 min ischemia and 40 min reperfusion. B: Ventricular sclices after triphenyltetrazoliumchloride (TTC) staining, placed between glass plates and transparent plastic grid placed on it. C: Representive ventricular sclices after TTC staining, placed between two glass plates and transparent plastic grid placed on it for calculation of percentage infarct size by volume method. D: Larger view of representive ventricular sclices after TTC staining, placed between two glass plates and transparent plastic grid placed on it for calculation of percentage infarct size by volume method. Yellow part represents infarcted part and pink part shows viable part after 20 min ischemia and 40 min reperfusion.

After the heart was removed from the Langendorff's apparatus, both the auricles and root of aorta were excised, and the ventricles were kept overnight at 4°C. The frozen ventricles were sliced into uniform sections of 2-3 mm thickness. The slices were incubated in 1% triphenyltetrazoliumchloride (TTC) at 37°C in 0.2 mol/L Tris buffer (pH 7.4) for 20 min. TTC is converted into red formazone pigment by NADH and dehydrogenase enzyme; therefore, the viable cells were stained a deep red. The infarcted cells have lost the enzyme and cofactor and thus remained unstained or a dull yellow. The ventricular slices were placed between two glass plates. A transparent plastic grid with 100 squares in 1 cm^2^ was placed over an average stained area of each ventricular slice is calculated by counting the number of squares on either side. Similarly, the number of squares falling over non-stained dull yellow areas was also counted. Infarcted area is expressed as a percentage of total ventricular area (***Fig. 1***).

### Experimental protocol

**Fig. 2:**
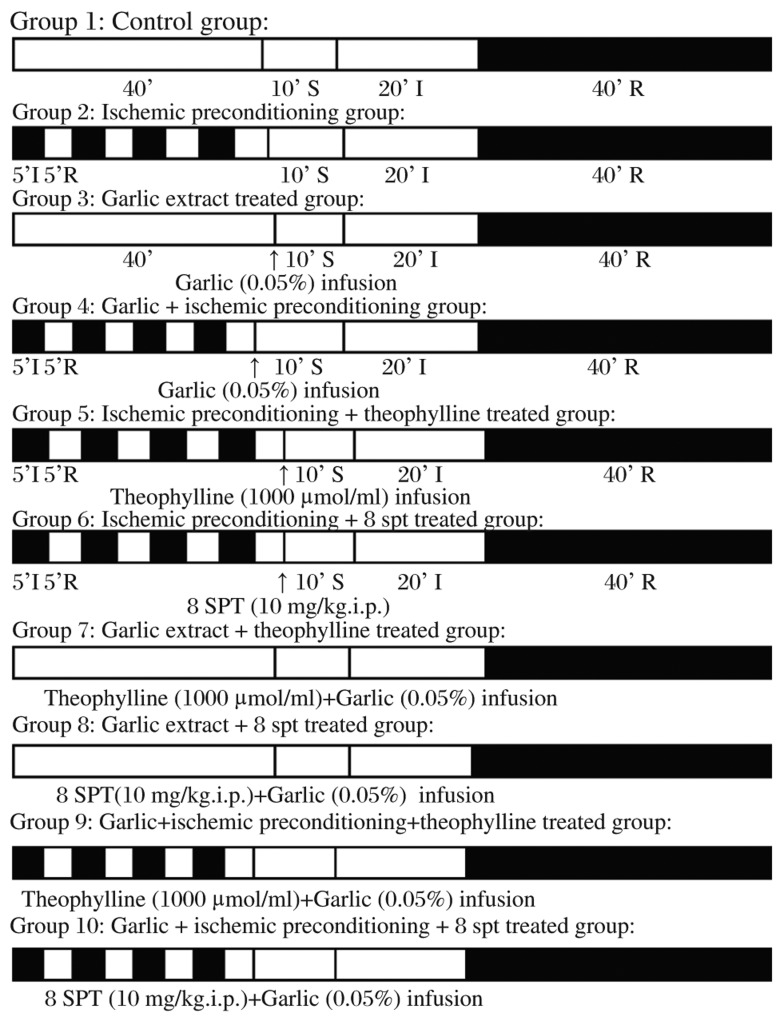
Diagrammatic representation of the experimental protocol. I, R, S denotes ischemia, reperfusion and stabilization, respectively.

Ten groups of Wistar albino rats were employed in the study (*n* = 6, for each group), and a total of 60 rats were used in this study (***Fig. 2***). Rats were subjected to surgical procedure for isolation of the left anterior descending coronary artery, with or without being subjected to the ischemic preconditioning protocol (i.e. four episodes of ischemia and reperfusion, 5 min each). Hearts were then excised immediately after operation and mouned on a Langendorff's apparatus and subjected to ischemia-reperfusion.

#### Group I: Control group (ischemia-reperfusion)

Rats were subjected to the surgical procedure for isolation of the left anterior descending coronary artery, and subjected to ischaemic preconditioning protocol. Then hearts were excised immediately after 40 min of sham operation and mounted on a Langendorff's apparatus and subjected to global ischemia for 20 min, followed by reperfusion for 40 min.

#### Group II: Ischemic preconditioning group

Rats were subjected to the surgical procedure for isolation of the left anterior descending coronary artery, and subjected to ischaemic preconditioning protocol. Then hearts were excised immediately and mounted on a Langendorff's apparatus and subjected to global ischemia for 20 min, followed by reperfusion for 40 min.

#### Group III: Garlic extract treated group

Rats were subjected to the surgical procedure for isolation of the left anterior descending coronary artery, but not subjected to ischemic preconditioning protocol. Then hearts were excised immediately after 40 min of sham operation and mounted on a Langendorff's apparatus and subjected to global ischemia for 20 min, followed by reperfusion of garlic extract (0.05%) for 40 min.

#### Group IV: Garlic+ischemic preconditioning group

Rats were subjected to the surgical procedure for isolation of the left anterior descending coronary artery, and subjected to ischemic preconditioning protocol and hearts were then excised immediately and mounted on a Langendorff's apparatus and subjected to global ischemia for 20 min, followed by reperfusion of garlic extract (0.05%) for 40 min.

#### Group V: Ischemic preconditioning+theophylline treatment group

Rats were subjected to the surgical procedure for isolation of the left anterior descending coronary artery, and subjected to the ischemic preconditioning protocol. Hearts were then excised immediately and mounted on a Langendorff's apparatus and subjected to global ischemia for 20 min, followed by reperfusion for 40 min of theophylline (1,000 µmol/L, highest dose employed to inhibit adenosine receptors)[31].

#### Group VI: Ischemic preconditioning+8-SPT treatment group

Rats were subjected to the surgical procedure for isolation of the left anterior descending coronary artery, and subjected to ischemic preconditioning protocol. 8-SPT (10 mg/kg, i.p.)[32] was given at the beginning of last reperfusion. Then hearts were excised immediately and mounted on a Langendorff's apparatus and subjected to global ischemia for 20 min, followed by reperfusion for 40 min.

#### Group VII: Garlic + theophylline treatment group

Rats were subjected to the surgical procedure for isolation of the left anterior descending coronary artery, and not subjected to ischaemic preconditioning protocol. Then hearts were excised immediately after 40 min of sham operation and then mounted on a Langendorff's apparatus and subjected to global ischemia for 20 min, followed by reperfusion of garlic extract (0.05%) and theophylline (1,000 µmol/L) for 40 min.

#### Group VIII: Garlic + 8-SPT treatment group

Rats were subjected to the surgical procedure for isolation of the left anterior descending coronary artery, and not subjected to ischaemic preconditioning protocol. 8-SPT (10 mg/kg, i.p.)[32] was given at the beginning of last reperfusion. Then hearts were excised immediately after 40 min of sham operation and then mounted on a Langendorff's apparatus and subjected to global ischaemia for 20 min, followed by reperfusion of garlic extract (0.05%) for 40 min.

#### Group IX: Garlic+ischemic preconditioning + theophylline treatment group

Rats were subjected to the surgical procedure for isolation of the left anterior descending coronary artery, and subjected to the ischemic preconditioning protocol. Then hearts were excised immediately after 40 min of sham operation and then mounted on a Langendorff's apparatus and subjected to global ischemia for 20 min, followed by reperfusion of garlic extract (0.05%) and theophylline (1,000 µmol/L) for 40 min.

#### Group X: Garlic+ischemic preconditioning + 8-SPT treatment group

Rats were subjected to the surgical procedure for isolation of the left anterior descending coronary artery, and subjected to ischemic preconditioning protocol. 8-SPT (10 mg/kg, i.p.)[32] was given at beginning of last reperfusion. Hearts were then excised immediately after 40 min of sham operation and then mounted on a Langendorff's apparatus and subjected to global ischemia for 20 min, followed by reperfusion of garlic extract (0.05%) for 40 min.

### Estimation of lactate dehydrogenase (LDH) and nitrite

LDH was estimated in coronary effluent by the 2,4-DNPH method as described by King[33]. Nitrite concentration in coronary effluent was estimated by Griess Reaction[34],[35].

### Analysis of coronary venous adenosine efflux

Coronary venous effluent was sampled every min for the first 5 min of reperfusion and then collected at 5, 15, 20, 30 and 40 min time points for the remainder of the reperfusion period. The samples were snap frozen at -80°C until they were analyzed by reverse phase HPLC. Adenosine in coronary venous effluent was analyzed by reverse phase HPLC (Younglin Instruments, Model-Acme 9000, 899-6 Hogye-dong, Anyang, 431-836, Korea) using 18′S column (Agela Technologies) for total post-ischemic purine efflux values. Purine nucleosides were detected at 254 nm, using external standards to quantify the compounds of interest.

### Statistical analysis

Values for enzymatic data and infarct size were expressed as mean±S.E.M. Statistical significance was calculated using one-way analysis of variance. Dunnett's test and Student's *t*-test were employed for comparison with the control group and for multiple comparisons between groups, respectively. A value of *P* < 0.05 was considered to be significant.

## RESULTS

### Effect of ischemic preconditioning and garlic extract, and 8-SPT and theophylline (adenosine inhibitor) reperfusion on heart rate and coronary flow rate

Global ischemia for 20 min produced significant decrease in heart rate and coronary flow rate, which persisted for the entire 40 min of reperfusion. Ischemic preconditioning and garlic extract (0.05%) reperfusion significantly increased heart rate and coronary flow rate. 8-SPT and theophylline reperfusion significantly decreased heart rate and coronary flow rate (***Table 1***, 2).

**Table 1:** Coronary flow rate in isolated rat heart subjected to global ischemia (20 min) and reperfusion (40 min).

Groups	Basal	5 min	10 min	20 min	40 min
Group I	8.40 ± 0.13	4.40 ± 0.24*	5.32 ± 0.05*	4.98 ± 0.18*	4.16 ± 0.42*
Group II	10.22 ± 0.30	4.36 ± 0.37*	4.94 ± 0.29*	4.30 ± 0.50*	4.92 ± 0.67*
Group III	11.38 ± 1.53	7.04 ± 0.72*	4.92 ± 0.72*	4.04 ± 0.71*	3.92 ± 0.73*
Group IV	10.73 ± 0.49	5.98 ± 0.49*	4.82 ± 1.06*	3.93 ± 1.12*	3.53 ± 0.20*
Group V	8.06 ± 0.63	4.78 ± 0.79*	3.98 ± 0.75*	3.70 ± 0.70*	2.58 ± 0.65*
Group VI	9.66 ± 0.96	6.86 ± 1.19*	6.46 ± 1.06*	6.00 ± 0.71*	5.20 ± 1.10*
Group VII	10.03 ± 0.59	5.79 ± 0.62*	5.96 ± 0.41*	5.06 ± 1.25*	4.17 ± 0.83*
Group VIII	9.51 ± 0.35	5.29 ± 0.45*	4.02 ± 0.37*	3.95 ± 0.48*	2.79 ± 0.63*
Group IX	9.67 ± 0.28	6.19 ± 1.02*	5.39 ± 1.02*	5.26 ± 0.63*	3.82 ± 0.63*
Group X	9.52 ± 0.69	7.03 ± 0.54*	6.08 ± 0.97*	5.49 ± 0.37*	4.94 ± 0.47*

Coronary flow rate was measured after stabilization (basal), 5,10, 20 and 40 min after reperfusion (R). Group I : control group (ischemia-reperfusion); Group II : ischeamic preconditioning group; Group III: garlic extract treatment group; Group IV : garlic + ischeamic preconditioning group; Group V: ischeamic preconditioning + theophylline treatment group; Group VI: ischeamic preconditioning + 8-SPT treatment group; Group VII: garlic + theophylline treatment group; Group VIII : garlic + 8-SPT treatment group; Group IX : garlic + ischeamic preconditioning + theophylline treatment group; Group X : garlic + ischemic preconditioning + 8-SPT treatment group; *n* = 6 for each group, *P < 0.05 *vs* control.

(mL/min, *x*±*s*)

**Table 2:** Heart rate in isolated rat heart subjected to global ischaemia (20 min) and reperfusion (40 min).

Groups	Basal	5 min	10 min	20 min	40 min
Group I	215 ± 30	224 ± 20*	194 ± 20*	186 ± 15*	192 ± 10*
Group II	228 ± 35	240 ± 30*	210 ± 30*	204 ± 30*	196 ± 30*
Group III	246 ± 18	256 ± 31*	234 ± 08*	222 ± 13*	213 ± 22*
Group IV	267 ± 22	302 ± 13*	278 ± 26*	243 ± 28*	240 ± 07*
Group V	212 ± 12	224 ± 23*	212 ± 15*	196 ± 15*	208 ± 20*
Group VI	216 ± 26	220 ± 19*	194 ± 17*	192 ± 08*	184 ± 10*
Group VII	228 ± 23	236 ± 22*	229 ± 18*	216 ± 26*	224 ± 06*
Group VIII	217 ± 24	222 ± 24*	216 ± 25*	212 ± 14*	201 ± 16*
Group IX	232 ± 19	247 ± 26*	227 ± 28*	221 ± 28*	219 ± 24*
Group X	221 ± 33	249 ± 31*	218 ± 32*	218 ± 21*	202 ± 27*

Heart rate was measured after stabilization (basal), 5, 10, 20 and 40 min after reperfucion (R). Group I : control group (ischemia-reperfusion); Group II : ischeamic preconditioning group; Group III : garlic extract treatment group; Group IV: garlic + ischemic preconditioning group; Group V: ischeamic preconditioning + theophylline treatment group; Group VI : ischeamic preconditioning + 8-SPT treatment group; Group VII: garlic + theophylline treatment group; Group VIII : garlic + 8-SPT treatment group; Group IX : garlic + ischeamic preconditioning + theophylline treatment group; Group X : garlic + ischemic preconditioning + 8-SPT treatment group; *n* = 6 for each group, *P < 0.05 *vs* control.

(beats/min, mean±SEM)

### Effect of ischemic preconditioning and garlic extract on ischemia-reperfusion induced myocardial infarct size

The extent of myocardial infarct size in control experiments was recorded to be (50.167±0.832)%, calculated by the volume method. The ischemic preconditioning and garlic treatment group showed significant decrease in myocardial infarct size, when compared to the control group. Garlic extract administered during ischemic preconditioning was found to significantly decrease the myocardial infarct size, when compared to ischemic preconditioning, thereby further exaggerating the decrease in infarct size caused by ischemic preconditioning (***Table 3***).

**Table 3:** Myocardial infarct size by volume method in isolated rat heart after subjected to global ischamia (20 min) followed by reperfusion (40 min).

Groups	Percentage infarct
Group I	50.167 ± 0.832
Group II	30.010 ± 0.787*
Group III	25.197 ± 1.061*
Group IV	16.480 ± 0.612*
Group V	34.687 ± 0.505*
Group VI	49.437 ± 1.061
Group VII	28.415 ± 0.373*
Group VIII	41.922 ± 1.020*
Group IX	23.597 ± 0.573*
Group X	37.845 ± 1.044

Group I : control group (ischemia-reperfusion); Group II : ischeamic preconditioning group; Group III: garlic extract treatment group; Group

IV: garlic + ischeamic preconditioning group; Group V : ischeamic preconditioning + theophylline treatment group; Group VI: ischeamic preconditioning + 8-SPT treatment group; Group VII: garlic + theophylline treatment group; Group VIII : garlic + 8-SPT treatment group; Group IX : garlic + ischeamic preconditioning + theophylline treatment group; Group X : garlic + ischeamic preconditioning + 8-SPT treatment group. *n* = 6 for each group,*P < 0.05 *vs* control.

(%,mean±SEM)

### Effect of theophylline and 8-SPT reperfusion on ischemic preconditioning and garlic extract on ischemia-reperfusion induced myocardial infarct size

Theophylline (1,000 µmol/L) and 8-SPT (10 mg/kg, i.p.) reperfusion significantly attenuated ischemic preconditioning. Garlic extract and garlic+ischemic preconditioning induced a decrease in myocardial infarct size (***Table 3***).

### Effect of preconditioning and garlic extract on ischemia-reperfusion induced LDH release

The extent of LDH release in coronary effluent at 15 min reperfusion in control experiments was recorded to be (838.000±1.897) U/L. The ischemic preconditioning and garlic treatment group showed significant decrease in LDH release, when compared to the control group. Garlic extract (0.05%) reperfused after ischemic preconditioning was found to significantly decrease the LDH release after global ischemia, when compared to ischemic preconditioning, thereby further exaggerating the decrease in LDH release caused by ischemic preconditioning (***Table 4***).

**Table 4:** Lactate dehydrogenase (LDH) release in coronary effluent of isolated rat heart at 15 min reperfusion, subjected to global ischemia (20 min) followed by reperfusion (40 min).

Groups	LDH(U/L)
Group I	838.000 ± 1.897
Group II	495.333 ± 6.146*
Group III	413.000 ± 4.333*
Group IV	276.167 ± 4.556*
Group V	627.667 ± 4.080*
Group VI	823.667 ± 4.469
Group VII	493.000 ± 5.404*
Group VIII	790.000 ± 7.233*
Group IX	700.167 ± 5.782*
Group X	769.723 ± 3.483

Group I: control group (ischemia-reperfusion); Group II : ischeamic preconditioning group; Group III: garlic extract treatment group; Group IV: garlic + ischeamic preconditioning group; Group V: ischeamic preconditioning + theophylline treatment group; Group VI : ischeamic preconditioning + 8-SPT treatment group; Group VII: garlic + theophylline treatment group; Group VIII: garlic + 8-SPT treatment group; Group IX : garlic + ischeamic preconditioning + theophylline treatment group; Group X: garlic + ischeamic preconditioning + 8-SPT treatment group. *n* = 6 for each group,*P < 0.05 *vs* control.

(IU/L, *x*±*s*)

### Effect of theophylline and 8-SPT reperfusion on ischemic preconditioning and garlic extract on ischemia-reperfusion induced LDH release

Theophylline reperfusion (1,000 µmol/L) and 8-SPT (10 mg/kg, i.p.) significantly attenuated ischemic preconditioning, and garlic extract and garlic+ischemic preconditioning induced a decrease in LDH release (***Table 4***).

### Effect of ischemic preconditioning and garlic extract on ischemia-reperfusion induced nitrite release

The extent of nitrite level in control experiments was recorded to be (521.000±1.283) µg(basic), (530.667±1.054) µg (immediately), (510±1.317) µg (40 min). The ischemic preconditioning and garlic extract (0.05%) treatment showed significant increase in nitrite release noted in basal, immediately and 40 min after reperfusion, when compared to the control group. Garlic extract administered after ischemic preconditioning was found to significantly increase the nitrite release after global ischemia, when compared to ischemic preconditioning, thereby further exaggerating an increase in nitrite release caused by ischemic preconditioning (***Table 5***).

**Table 5:** Nitrite release at basal, immediate (5 min reperfusion) and 40 min reperfusion in coronary effluent of isolated rat heart subjected to global ischemia (20 min) followed by reperfusion (40 min)

Groups	Time point	Nitrite release (µg/mL)
Group I	Basal	521.000 ± 1.238
	Immediate reperfusion	530.667 ±1.054
	40 min reperfusion	510.000 ± 1.317
Group II	Basal	669.167 ±1.046*
	Immediate reperfusion	701.000 ± 1.390*
	40 min reperfusion	650.500 ± 0.992*
Group III	Basal	860.333 ± 13.288*
	Immediate reperfusion	866.833 ± 13.833*
	40 min reperfusion	838.167 ± 12.081*
Group IV	Basal	956.333 ± 6.393*
	Immediate reperfusion	972.167 ± 2.931*
	40 min reperfusion	963.573 ± 4.321*
Group V	Basal	480.000 ± 1.033*
	Immediate reperfusion	490.000 ± 1.033*
	40 min reperfusion	480.833 ± 0.703*
Group VI	Basal	420.333 ± 5.649*
	Immediate reperfusion	448.667 ± 5.283*
	40 min reperfusion	446.167 ± 17.895*
Group VII	Basal	439.500 ± 1.232*
	Immediate reperfusion	489.833 ± 0.910*
	40 min reperfusion	471.000 ± 1.390*
Group VIII	Basal	547.333 ± 17.795*
	Immediate reperfusion	564.000 ± 14.656*
	40 min reperfusion	541.167 ± 13.152*
Group IX	Basal	421.667 ± 1.810*
	Immediate reperfusion	469.667 ± 0.989*
	40 min reperfusion	441.000 ± 1.713*
Group X	Basal	614.000 ± 5.944*
	Immediate reperfusion	671.667 ± 5.277*
	40 min reperfusion	635.833 ± 5.588*

Group I : control group (ischemia-reperfusion); Group II : ischeamic preconditioning group; Group III : Garlic extract treatment group; Group IV: garlic + ischeamic preconditioning group; Group V: ischeamic preconditioning + theophylline treatment group; Group VI: ischeamic preconditioning + 8-SPT treatment group; Group VII : garlic + theophylline treatment group; Group VIII : garlic + 8-SPT treatment group; Group IX : garlic + ischeamic preconditioning + theophylline treatment group; Group X: garlic + ischeamic preconditioning + 8-SPT treatment group, *n* = 6 for each group,*P < 0.05 *vs* control.

(µg/mL, *x*±*s*)

### Effect of theophylline and 8-SPT treatment on ischemic preconditioning and garlic extract on ischemia-reperfusion induced nitrite release

Theophylline reperfusion (1000 µmol/L) and 8-SPT (10 mg/kg, i.p.) significantly attenuated ischemic preconditioning, and garlic extract and garlic+ischemic preconditioning induced an increase in nitrite release (***Table 5***).

### Effect of ischemic preconditioning and garlic extract on ischemia-reperfusion induced adenosine release in coronary effluent

The extent of adenosine level in control experiments was recorded to be (630.333±7.219) µmol/mL (line), (662.000±4.829) µmol/mL (immediately), (647.000±1.483) µmol/mL (40 min). Ischemic preconditioning and garlic extract (0.05%) treatment showed significant increase in adenosine release noted in basal, immediately and 40 min after reperfusion, as compared to the control group. Garlic extract administered after ischemic preconditioning was found to significantly increase the adenosine release after global ischemia, when compared to ischemic preconditioning, thereby further exaggerating the increase in adenosine release caused by ischemic preconditioning (***Table 6***).

**Table 6:** Adenosine release at basal, immediate (5 min reperfusion) and 40 min reperfusion in coronary effluent of isolated rat heart subjected to global ischemia (20 min) followed by reperfusion (40 min).

Groups	Time point	Nitrite release (µg/ml)
Group I	Basal	630.333 ± 7.219
	Immediate reperfusion	662.000 ± 4.829
	30 min reperfusion	647.000 ± 1.483
Group II	Basal	757.000 ± 1.461*
	Immediate reperfusion	769.500 ± 7.575*
	30 min reperfusion	745.167 ± 2.833*
Group III	Basal	1005.333 ± 13.032*
	Immediate reperfusion	1035.333 ± 3.422*
	30 min reperfusion	1022.667 ± 3.870*
Group IV	Basal	1231.749 ± 5.832*
	Immediate reperfusion	1247.034 ± 4.824*
	30 min reperfusion	1243.082 ± 6.829*
Group V	Basal	584.833 ± 4.542
	Immediate reperfusion	628.500 ± 2.643
	30 min reperfusion	615.333 ± 3.393
Group VI	Basal	517.667 ± 4.631
	Immediate reperfusion	530.833 ± 3.341
	30 min reperfusion	521.333 ± 4.958
Group VII	Basal	557.667 ± 5.011
	Immediate reperfusion	571.000 ± 4.050
	30 min reperfusion	566.167 ± 2.386
Group VIII	Basal	808.000 ± 4.203
	Immediate reperfusion	829.500 ± 2.592
	30 min reperfusion	820.333 ± 3.313
Group IX	Basal	534.667 ± 3.095
	Immediate reperfusion	565.333 ± 5.004
	30 min reperfusion	551.000 ± 4.531
Group X	Basal	844.833 ± 3.637
	Immediate reperfusion	862.000 ± 4.865
	30 min reperfusion	857.333 ± 2.871

Group I : control group (ischemia-reperfusion); Group II : ischeamic preconditioning group; Group III: Garlic extract treatment group; Group IV : garlic + ischeamic preconditioning group; Group V : ischeamic preconditioning + theophylline treatment group; Group VI: ischeamic preconditioning + 8-SPT treatment group; Group VII: garlic + theophylline treatment group; Group VIII: garlic + 8-SPT treatment group; Group IX : garlic + ischeamic preconditioning + theophylline treatment group; Group X: garlic + ischeamic preconditioning + 8-SPT treatment group. *n* = 6 for each group,*P < 0.05 *vs* control.

(µmol/mL, *x*±*s*)

### Effect of theophylline and 8-SPT treatment on ischemic preconditioning and garlic extract on ischemia-reperfusion induced nitrite release

Theophylline reperfusion (1,000 µmol/L) and 8-SPT (10 mg/kg, i.p.) significantly attenuated ischemic preconditioning, and garlic extract and garlic+ischemic preconditioning induced an increase in adenosine release (***Table 6***).

### Toxic effect of high doses of garlic on isolated rat heart

**Fig. 3:**
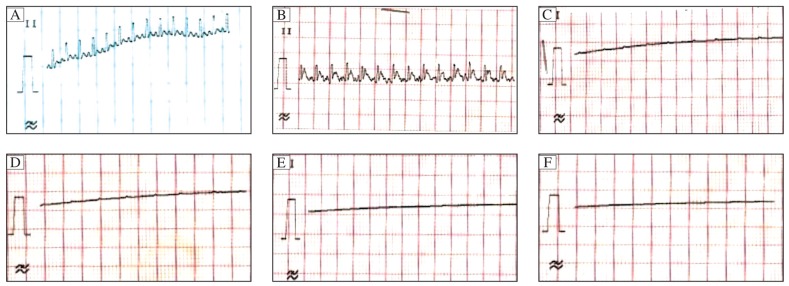
Electrocardiogram of rats treated with higher doses of aqueous extract of garlic (0.5%, 0.4%, 0.3%, 0.2%, 0.1% and 0.07%) showed arrhythmic, depressed heart rate (cardiodepressant) and mortality at last on 40 min reperfusion. ECG taken in limb lead-II, on isolated heart, mounted on a Langendorff's appratus. A: Group I , control group. B: Group II , dose 0.1% of garlic aqueous extract (at 5 min reperfusion) found arrhythmic. C: Groups III, dose 0.1% of garlic aqueous extract (at 15 min reperfusion) found arrhythmic and cardio-depressant. D: Groups IV, dose 0.1% of garlic aqueous extract (at 20 min reperfusion) found arrhythmic and cardio-depressant. E: Groups V, dose 0.1% of garlic aqueous extract (at 30 min reperfusion) found complete cardio-depressant. F: Groups VI, dose 0.1% of garlic aqueous extract (at 40 min reperfusion) found complete cardio-depressant and mortality.

This study demonstrated that doses higher than 0.05% were cardio-toxic because they caused arrhythmia and cardio-depression and a significant decrease in heart rate whereas the lower doses produced an insignificant effect (***Fig. 3***).

## DISCUSSION

It has been reported that garlic has produced cardio-depressant and anti-arrhythmic actions. However the involvement of calcium in cardio-depressant action of garlic has been ruled out[36]-[38]. It has also been reported that garlic shows cardioprotective effect which is similar to ischemic preconditioning and that it is effective in reducing myocardial infarct size in an isolated rat heart[16]. Adenosine is important, both as a potential trigger and also as a mediator during the sustained ischemia. Adenosine may attenuate ischemia/reperfusion injury *via* a number of possible mechanisms, which include purine salvaging, improved tissue perfusion, anti-inflammatory action and a direct intracellular initiator/effector mechanism[10]. Adenosine has been involved in ischemic preconditioning and has been shown to have a cardioprotective effect. So, the present study was designed to standardize the cardioprotection offered by garlic and to evaluate the mechanism of action and role of adenosine in garlic cardioprotection.

In this investigation, we observed that reperfusion of theophylline (1,000 µmol/L, the highest dose employed to block adenosine receptors)[31] or 8-SPT (10 mg/kg, i.p.[32], nonselective adenosine receptor antagonist) increases the infarct size in rat subjected to ischemic preconditioning as well as in the garlic extract and garlic extract plus ischemic preconditioning group.

LDH is a known marker of cardiac injury and the release of LDH observed in this study occurred immediately and 40 min after reperfusion[39]. It may apparently be suggested that initial release of LDH occurring immediately after reperfusion may be due to ischemic injury and the delayed release of LDH, observed after 40 min of reperfusion, may be due to reperfusion injury. This is also in conformity with the earlier reports[40]-[42]. Ischemia/reperfusion injury has also been quantified by measuring the myocardial infarct size by the volume method[43].

The protocol of four episodes of 5 min ischemia interspersed with four episodes of 5 min reperfusion, employed in the present study, has been documented to precondition the myocardium. Since the original observation in an *in vivo* rabbit model, that the adenosine receptor blockers 8-SPT and PD115,199 were able to abolish cardioprotection afforded by ischemic preconditioning, the role of adenosine in ischemic preconditioning has been confirmed in other species such as dogs[44] and swine[45],[46], rats[47], and rabbits[48].

In previous studies, the interstitial adenosine concentration increased no earlier than after 5 min of ischemia and the interstitial adenosine concentration during the sustained ischemia was attenuated in the 3-min and 10-min ischemic preconditioning groups[49]. Attenuation of the increase in the interstitial adenosine concentration was also observed when ischemic preconditioning was abolished by adenosine receptor blockade, and therefore, it is not a reflection of the cardioprotective effect[50]. So in this study we have used mild stimulus of 5 min ischemia and 5 min reperfusion to test the hypothesis that the adenosine receptor is involved in garlic cardioprotection. In rat hearts, adenosine involvement in preconditioning has been positively reported in several isolated global ischemia models and intact regional ischemia models. However, the duration of preconditioning and endpoints to assess myocardial damage in the various studies have produced conflicting results regarding the involvement of adenosine in rat hearts[51].

In this study, the role of ischemic preconditioning in cardioprotection was evaluated and mediated *via* adenosine receptor activation, which has been well demonstrated in isolated rat heart models. Ischemic preconditioning showed significant decrease in myocardial infarct size and LDH level and a significant increase in coronary flow rate, heart rate, nitrite release and adenosine release. Theophylline and 8-SPT administration attenuates the benefits of ischemic preconditioning and garlic cardioprotection in this study. This result is contrary to literature data by Ganote and Armstrong[52], in which it was concluded that adenosine does not involve preconditioning of rat hearts because adenosine receptor antagonists could not attenuate the cardioprotective effects of ischemic preconditioning.

The higher interstitial adenosine in rat hearts than that in other species might be one of the reasons that 8-SPT could not abolish the cardioprotection of ischemic preconditioning[53],[54]. The short half-life of 8-SPT (about 10 min) might be another reason for the failure to abolish the protection when it was intravenously injected as in the study reported by Li and Kloner[55].

Theophylline, the adenosine antagonist used in this study, is known to act as a nonselective adenosine receptor antagonist, antagonizing A1, A2, and A3 receptors almost equally[12]. It also act as a competitive nonselective phosphodiesterase inhibitor, which raises intracellular cAMP, activates PKA, inhibits TNF-α and inhibits leukotriene synthesis, and reduces inflammation and innate immunity. In many studies theophylline has been used to evaluate the role of adenosine receptors[12],[56]-[61].

Previously, it has been shown that garlic extract can prevent ischemia-reperfusion induced myocardial injury, and has similar effects, such as ischemic preconditioning[16]. So, we have assumed that because garlic exhibits similar cardioprotection to ischemic preconditioning, it may be possible that the mechanism behind garlic cardioprotection involves adenosine similar to the mechanism of ischemic preconditioning.

Many chemical constituents present in garlic have been found to have a cardioprotective effect against IR injury and myocardial infarct size. Diallyl disulfide was found to protect the liver from warm IR injury by reducing oxidative stress[62]. *S*-allyl lcysteine diminishes cerebral ischemia-induced mitochondrial dysfunctions in the hippocampus[63] and was found to be protective in myocardial infarction *via* an H_2_S-related pathway[1]. Allicin has been shown to have protective effects on cerebral ischemia-reperfusion injuries[64]. Studies have shown that alicor (a marketed prepration of garlic) is effective in reducing multifactorial risk of cardiovascular diseases including atherosclerosis and MI[65],[66].

An investigation of the effects of a garlic dialysate on diastolic blood pressure (DBP), heart rate (HR) and electrocardiogram (ECG) of anaesthethized dogs and its effects on the frequency and tension of isolated rat atria was performed. Garlic dialysate led to a drop in DBP from (112.50±3.67) mmHg to (70.00±3.16) mmHg and a decrease in HR from (198.00±9.81) beats/min to (164.00±16.59) beats/min in a dose-dependent manner. The ECG showed a regular sinus bradycardic rhythm. The addition of garlic dialysate to the isolated left rat atria evoked a decrease in tension development. Frequency, measured by the spontaneous beating of the right atria, was also reduced. Both effects were dose-dependent. In addition to these effects, the positive inotropism and chronotropism induced by addition of isoproterenol 10^-9^ mol/L, were partially antagonized by preincubation of the rat atria with the garlic dialysate. The above findings can be explained by a depressant effect on automaticity and tension development in the heart, suggesting a β-adrenoceptor blocking action produced by the garlic dialysate[36]. The effects of garlic (*Allium sativum* L., Liliaceae) dialysate were studied on arrhythmias induced in anaesthetized dogs and on isolated left rat atria. Garlic dialysate suppressed premature ventricular contractions (PVC) and ventricular tachycardia (VT) in ouabain-intoxicated dogs as well as the ectopic rhythms induced by isoprenaline (10^-6^ mol/L) and aconitine (10^-8^ mol/L) on electrically driven left rat atria. The effective refractory period (ERP) and the sinus node recovery time (SNRT) of isolated rat atria were prolonged in a dose-dependent manner by the administration of this extract. Garlic dialysate decreased the positive inotropic and chronotropic effects of isoprenaline in a concentration-dependent manner. These last effects were increased by propranolol. The results suggest that garlic dialysate has a significant anti-arrhythmic effect in both ventricular and supraventricular arrhythmias[37]. Garlic oil is cardioprotective against isoprotenol induced rat myocardial necrosis in rats[67]. Makheja *et al*[68] in 1990 identified three main antiplatelet constituents, namely adenosine, allicin and paraffinic polysulfides in both garlic and onion. The study indicated that the observed *in vivo* antiplatelet effects of ingesting onion and garlic were attributable more to the adenosine than to the allicin and paraffinic polysulfide constituents. Aqueous extracts of fresh garlic (*Allium sativum* Linn.) inhibited efficiently the activity of adenosine deaminase (ADA) of cultivated endothelial cells[69].

In one study, comparison of the cardioprotective effects of freshly crushed garlic vis-avis that of processed garlic was done. Two groups of rats were gavaged with respective garlic preparations while the control group received vehicle only. After 30 days, all of the rats were sacrificed and isolated the hearts were subjected to 30 min ischemia followed by 2 h of reperfusion. Both of the garlic preparations provided cardioprotection, but superior cardiac performance was noticed for those fed with freshly crushed garlic. Consistent with these results, the freshly crushed garlic group displayed significantly greater phosphorylation of antiapoptotic ERK1/2 proteins, reduced Bax/Bcl-2 ratio, and reduced phosphorylation of proapoptotic p-38 MAPK and JNK. Moreover, the survival signaling network consisting of Akt-FoxO1 was increased in the freshly crushed garlic treated hearts. Freshly crushed garlic, but not the processed garlic, showed enhanced redox signaling as evidenced by an increased level of p65 subunit of NF-κB, Nrf2, and enhanced GLUT 4, PPAR-α, and PPAR-δ. The results thus show that although both freshly crushed garlic and processed garlic provide cardioprotection, the former has additional cardioprotective properties presumably due to the presence of H_2_S[70]. So in the present study freshly crushed garlic was used at a different dose level.

Aqueous extract of garlic was used in this study and the dose was standardized by comparing the effects of different doses of garlic on the isolated rat heart. Among the different doses, the 0.05% dose was found to be most effective.

It was also found that the cardioprotection provided by garlic and ischemic preconditioning was more effective than the ischemic preconditioning or garlic extract alone. This was demonstrated by the decrease in myocardial infarct size and LDH level and increased nitrite and adenosine levels in the coronary effluent. These findings supported the earlier finding that garlic acts as a stimulus to the cardioprotective effect of ischemic preconditioning.

In conclusion, we demonstrated that only a 0.05% concentration of garlic was cardioprotective, among several concentrations. The guideline for selecting the garlic dose was a dose higher than 0.05% showed (1) arrhythmia at 5 min reperfusion, (2) depressed heart rate (cardiodeprassion) at 15 min, 20 min, 30 min reperfusion and (3) mortality at the end of 40 min reperfusion. Arrhythmia or depressed function was also reflected by the extent of myocardial ischemia-reperfusion injury (i.e. higher percentage infarct size of 60% to 70%). The role of adenosine in garlic cardioprotection was ruled out in this study because garlic cardioprotection was significantly reduced by theophylline (1,000 µmol/L) and 8-SPT(10 mg/kg, i.p.) as expressed in terms of increased myocardial infarct size, increased LDH level and reduced nitrite and adenosine levels in the coronary effluent. Therefore, the cardioprotective effect of garlic extract was mediated through the adenosine receptors. It may be due to the chemical constituent-adenosine present in the garlic or due to the ability of garlic to inhibit adenosine deaminase. These findings suggest the role of adenosine and its receptors in the pharmacological and molecular mechanistic involvement in garlic cardioprotection. However the role of specific adenosine receptor subtype needs further research. Adenosine induced garlic cardioprotection was mediated by modulation of nitric oxide because nitrite and adenosine, both increase in garlic cardioprotection.
